# Thoracic aortic size in Brazilian smokers: measures using low-dose chest computed tomography anatomical and epidemiological assessment

**DOI:** 10.6061/clinics/2021/e2315

**Published:** 2021-01-11

**Authors:** Lucas Lembrança, Marcelo Passos Teivelis, Adriano Tachibana, Ricardo Sales dos Santos, Richard Wonuh Joo, Emanuela Zippo, Nelson Wolosker

**Affiliations:** Hospital Israelita Albert Einstein, Sao Paulo, SP, BR

**Keywords:** Aorta, Diameter, Arch, Thoracic

## Abstract

**OBJECTIVES:**

Thoracic aortic aneurysms (TAAs) represent one-third of the hospitalizations for aortic diseases. The prevalence rate depends on the definition of the normal size of the aorta, which is quite variable, depending on the population studied. The aim of this study was to evaluate the characteristics of the thoracic aorta of Brazilian smokers, identifying the normal size of the aorta, presence of anatomical variations, and prevalence of TAA.

**MATERIALS AND METHODS:**

A total of 711 patients underwent radiological evaluation with low-dose computed tomography (CT) from January 2013 to July 2014 with the initial objective of lung nodule tracking. Two examiners evaluated these images, and measurements of maximum and serial diameters were performed manually in true orthogonal planes. Serial diameter measurements were taken every 2 cm in the ascending aorta and 5 cm in the descending segment. We searched for anatomical variations, aortic arch type, and correlations between anatomical characteristics, sex, body mass index, and body surface area (BSA).

**RESULTS:**

The maximum diameters were 33.61 (standard deviation [SD] 3.88), 28.66 (SD 2.89), and 28.36 mm (SD 3.09) for the ascending segment, aortic arch, and descending segment, respectively. A positive correlation was found between male sex, age, and BSA and aorta diameter. The bovine arch was the most common variation of the aortic arch type, and we found one (0.14%) case of TAA.

**CONCLUSIONS:**

This study with low-dose CT allowed the determination of the mean diameters of the ascending aorta, aortic arch, and descending aorta in Brazilian smokers and TAA prevalence.

## INTRODUCTION

Aortic aneurysms are the third leading cause of vascular disease in the United States. Thoracic aortic aneurysms (TAAs) represent one-third of the hospitalizations for aortic diseases (1). TAAs are classified according to their anatomical location: ascending aorta aneurysms (most common), aortic arch aneurysms, descending TAAs, and thoracoabdominal aneurysms (2).

TAAs and infrarenal abdominal aortic aneurysms share the same risk factors: smoking, systemic hypertension, and dyslipidemia (3). TAAs are often asymptomatic, which hinders their diagnosis, present slow growth, are strongly related to congenital and degenerative diseases (4), and can cause severe complications such as upper limb ischemia, carotid and vertebral embolization, and, the most feared of all, rupture.

The surgical treatment of aneurysms aims to reduce the risk of these complications and to treat symptoms, when they exist, to avoid rupture. However, surgical treatment is not risk-free (5,6). Thus, it is only recommended when the maximum diameter of the aneurysm, in the descending thoracic aorta, is greater than 6 cm (7). Above these diameters, the risks of surgery are lower than the risk of rupture, justifying treatment.

Several studies have sought to identify the actual incidence of TAAs; however, the incidence rate depends on the definition of the normal size of the aorta, which is quite variable, depending on the population studied. Hence, it is important to first determine the characteristics of a normal population to evaluate the incidence of TAAs. Recently, two studies were conducted to analyze the average size of all segments of the thoracic aorta using chest computed tomography (CT) in patients without risk factors for TAAs and thus evaluate the prevalence of TAAs in these populations (8,9). Itani et al., in Japan, in 2002 (8), used the population’s standard deviation (SD) to define an abnormality value; values above three SDs were considered abnormal. Thus, they found a prevalence of 0.16%. Kälsch et al., in 2013, in Germany, defined any dilatation of the thoracic aorta with a diameter greater than 5 cm as a TAA, resulting in a prevalence of 0.34% (9).

In Brazil, this type of study has not yet been performed and is relevant both internally to demonstrate the local anatomical conditions and to the rest of the world to demonstrate the difference in relation to other countries and the possible need for individualized population studies.

The aim of this study was to evaluate the characteristics of the thoracic aorta of Brazilian smokers, identifying the normal size of the aorta, presence of anatomical variations in the supra-aortic trunks, and prevalence of TAAs.

## MATERIALS AND METHODS

A total of 711 volunteers aged between 55 and 74 years, smokers (tobacco use 30 packs/year or more), and without signs or symptoms of aortic diseases were studied. All patients underwent radiological evaluation with low-dose CT from January 2013 to July 2014 with the initial objective of lung nodule tracking. Some of them were followed up, but at this moment, we analyzed only the first images. The anthropometric characteristics are presented in Table 1. The included patients were in the sixth decade of life and had mild overweight and a similar distribution between sexes, with an average body surface area (BSA) of 1.83 m^2^.

The tests were performed using a single device (Toshiba Aquilion 64 CFX CT, Toshiba Medical Systems Corporation, Otawara, Tochigi, Japan) and a low radiation dose technique (120 kV and a maximum of 15 mA with a 1×1 mm collimation). The images were stored in a Picture Archiving and Communication System (PACS) in the Digital Imaging and Communications in Medicine (DICOM) format.

These images were evaluated by two examiners, and measurements were performed manually using Horos version 3.2.0 (Horos Project, Geneva, Switzerland). At each segment, diameter measurements were taken of the thoracic aorta (ascending aorta, aortic arch, and descending aorta) in true orthogonal planes, corrected by the central axis, by a single examiner.

Then, serial measurements were made at precisely defined points. In the ascending aorta, diameter measurements were taken every 2 cm with the origin immediately proximal to the brachiocephalic trunk. In the descending aorta, serial measurements were performed every 5 cm with the origin immediately distal to the origin of the left subclavian artery.

**Figure 1:**
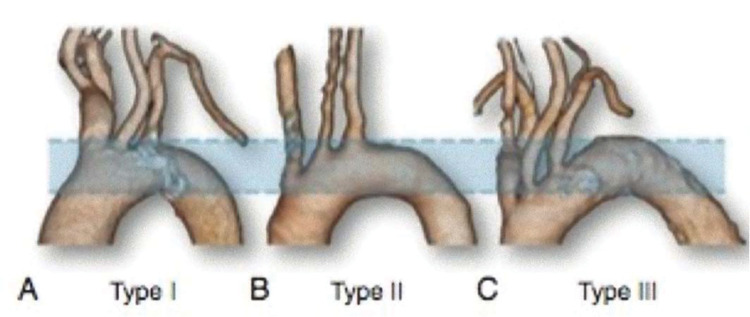
Aortic arch classification.

The most frequent anatomical variations were evaluated: the presence of a common origin between the right carotid artery and the brachiocephalic trunk (bovine arch) and the presence of an abnormal origin of the left vertebral artery and aberrant left subclavian artery. Aortic arch types were classified as follows: type 1, when the origin of the supra-aortic trunk is found in an imaginary plane above the greater curvature of the aorta; type 2, when one of the vessels originates in a plane between the greater and lesser curvature of the aorta; and type 3, when one of the vessels originates proximal to the plane drawn in the lesser curvature of the aorta (Figure 1). In addition, anatomical characteristics were correlated with anthropometric characteristics, body mass index (BMI), and BSA.

To identify the importance of each variable in determining the diameter value for each segment, we performed multiple linear regression with the following parameters: sex, age, and average BSA. Based on this analysis, we obtained the expected measures for each segment, stratified by sex and age group.

This study was approved by the institution’s ethics committee and exempt from informed consent.

## RESULTS

For each patient, we performed, at least, six measures of aortic diameters: two in the ascending segment, one in the aortic arch, and three in the descending aorta. This resulted in 4,266 diameter measurement points and a total of 8,532 measurements.

**Figure 2:**
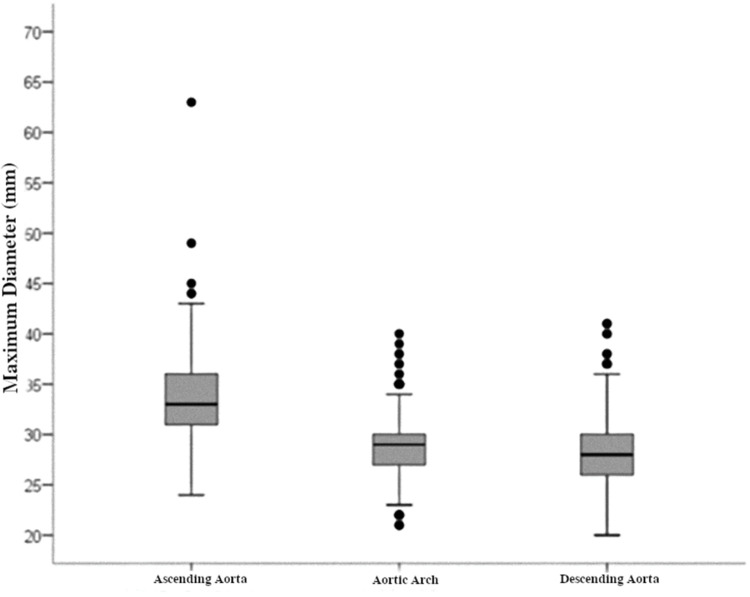
Aortic diameters by segment.

The maximum diameters of each segment are shown in Figure 2. The largest arterial diameter was observed in the ascending segment, at 33.61 mm (SD, 3.88). In the aortic arch and the descending aorta, we found smaller diameters (28.66 mm [SD 2.89] and 28.36 [SD 3.09], respectively).

Analysis of the correlation between values and anthropometric characteristics showed a positive correlation between male sex, age, and BSA with aorta diameter, with a stronger correlation for BSA and the descending aorta (Table 2).

**Figure 3:**
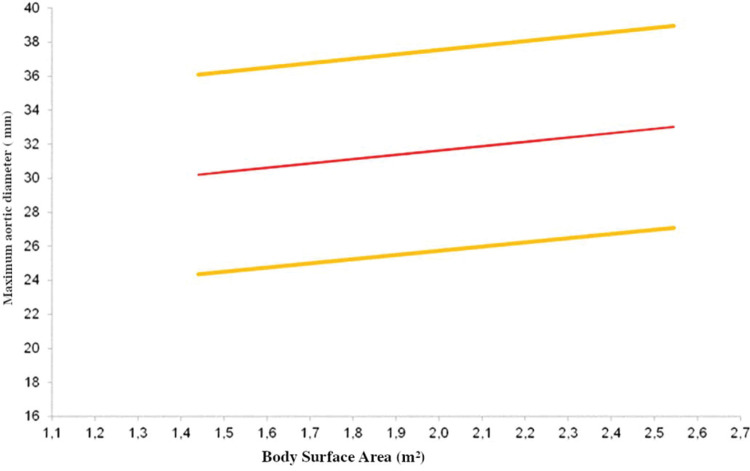
Expected descending aorta diameter for body surface area.

Multiple linear regressions confirmed that age, BSA, and male sex were good predictors of aortic size in the descending segment, as shown in Table 3. The equations used to calculate the diameter of each segment are shown in Table 4, and the expected aortic diameters for each segment are represented in Figure 3.

Anatomical variations were observed in 81 (11.4%) patients, and the bovine arch was the most common variation. Among the aortic arch types, type 1 was the most common (Table 5).

Finally, we found only one (0.14%) aneurysm in the descending aorta.

## DISCUSSION

TAA is a silent disease of increasing incidence and high morbidity and mortality, the prognosis of which depends substantially on the size of the aorta in each segment. In the past, analyses of normal vessel dimensions were performed based on angiography, ultrasound (10), and magnetic resonance imaging (11). With the increased availability of tomography, studies to identify the anatomical features of the aorta have been performed. In recent years, several studies have been conducted with this aim in North America (2,12,13), (14) Europe (9), and the East (8,15). The objective of our study was to analyze the dimensions of the aorta in Brazil and evaluate the impact of BMI, BSA, and age.

Currently, there is increasing concern about the deleterious effects of radiation. Thus, the use of tomography exams with low radiation doses, as performed in our study, is gaining importance. Wolak et al. (12) were the first to evaluate, on a large scale, the dimensions of large vessels with the use of tomography without contrast. Their data were comparable to those of a contrasted series and showed a high interobserver correlation. Thus, we can infer that the modern protocols do not require contrast and high doses of radiation to measure aortic diameters.

In our study, we used a sample previously selected for the screening of lung diseases (16). Thus, our study included patients in an older age group, similar to two studies conducted in Japan and Korea, which also used samples previously selected for lung neoplasia screening (8,15).

The demographic characteristics of our sample were similar to those in a national study that evaluated causes of death in Brazilian patients with aortic aneurysms (17), with no predominance of any sex and with a slightly higher than average age than American (2) and German (9) populations.

The maximum diameter of the ascending aorta was similar to that reported for two American series that used patients undergoing coronary disease control exams (12,18) and was lower than that reported for a large population study (2). This difference could be due to selection bias. A review which compared the works of Rogers, Wolak and Mao enrolled younger patients who consequently presented with fewer comorbidities and smaller vessels.

When compared with the mean diameter of the ascending aorta in non-American populations, the mean diameter of the ascending aorta in Brazilians was 1 mm greater than that found in Japanese patients (8) (younger patients) and 5 mm smaller than that found in a German population (9).

The aortic arch diameter tends to be less evaluated in series because its evaluation demands a more arduous methodology. Even so, we could compare our results with those reported in a study by Hager et al. (19) in German patients, which showed a slightly higher average diameter than that in Brazilians. However, the diameter of the descending aorta was higher than that reported by the majority of series (8,9,12,15), except for the Japanese series (8), which can be explained by the mean age of our patients, which was 60.9 years, compared to other studies with populations younger than 60 years (9,19).

Therefore, the diameter of the ascending aorta was smaller in Europeans and larger in Asians, while the diameter of the descending aorta had opposite characteristics.

There was a significant difference in measurements between sexes, with larger diameters in men in all segments, based on data previously demonstrated by other studies (9,12) and reaffirmed in our study.

The relationship between anthropometric data and vessel dimensions is already known. BMI, age, and BSA were related to the diameter of the thoracic aorta, with correlations similar to European and Asian studies. Thus, we sought to correlate aortic diameters with BMI and mean BSA, which is more accurate than BMI in evaluating the correlation between body measurements and aortic diameters (20).

Our patients had a mean BMI of 26.28, which is compatible with Brazilian data, which show a high incidence of overweight status, especially in age groups older than 60 years. The population in our study showed a lower BSA than most studies (9,12,19), with the exception of a Korean study (15). These variations confirm the need for careful evaluation when comparing data from different populations.

Keeping in mind that our population has a smaller BSA than others and that there was a correlation between BSA and aortic diameter in three segments (ascending aorta, trunk, and descending aorta), one would expect proportionally smaller diameters compared to those reported in other studies. We think that the high rate of smoking and the consequences of its use on the arterial wall led to an increase in aortic diameter.

The diameter is also influenced by age, which showed a significant correlation in all segments, especially in the descending aorta, as observed by Rogers et al. and Itani et al. (2,8). We know that there is a greater concentration of elastic tissue in the thoracic aorta as it approaches the diaphragm. Over time, degeneration of these fibers occurs, which leads to a progressive increase in the vessel by losing its elastic characteristics, becoming less compliant.

Because there is considerable phenotypic variation among the populations (Europeans, for example, have greater stature than Eastern populations), we performed multiple linear regression that resulted in a formula to find the expected diameter of each aortic segment as a function of BSA, BMI, and age range.

We observed an incidence of anatomical variations in the supra-aortic trunks in 11.4% of the sample. As in other studies (21,22), we found that the most common variation was the bovine arch, present in 9.6% of our sample. Mylonas (23) found an increased risk of aortic diseases in the presence of a bovine trunk; however, we found no relationship between anatomical variation and the presence of aneurysms.

Our study has some limitations. We are the first group to accurately assess the anatomical features of the aorta of Brazilians. These analyses were performed in non-contrast low-dose exams, which resulted in lower morbidity. In contrast to other series, which made two measurements in the axial plane (8,9,12,18), we used precisely defined sites in several aortic segments and performed all corrective measurements for the true axis of the vessel, which is the most accurate method (24) and recommended by the guidelines.

This resulted in 4,200 evaluated diameters that led us to find, with great accuracy, the real diameter of each segment (ascending aorta, aortic arch, and descending aorta).

In addition, the sample had a high prevalence of smokers and elderly individuals, which are factors usually related to larger aortic dimensions. However, they are the most frequent patients with TAAs and are therefore a good indicator of the measurements of patients at risk for developing the disease. In addition, the evaluation of the proximal segment of the aorta close to the sinus, an area of high tissue density that was impaired by the absence of contrast, prevented accurate measurements of the extent of the ascending aorta segment.

We believe that with technological evolution, using artificial intelligence and robotic analysis mechanisms, we will be able to evaluate large volumes of CT data and have more significant anatomical data from global populations in the near future.

## CONCLUSIONS

In elderly Brazilian smokers, who are part of a population at greatest risk for aneurysms, the mean diameters of the ascending aorta, aortic arch, and descending aorta were 33.6 mm, 28.7 mm, and 28.4 mm, respectively.

Anatomical variations were observed in 81 (11.4%) patients, with bovine arch being the most common, and TAA prevalence was 0.14%.

## AUTHOR CONTRIBUTIONS

Lembrança L, Teivelis MP, Tachibana A, dos Santos RS were responsible for the research, manuscript development, data collection and manuscript writing. Joo RW and Zippo E were responsible for the data collection. Wolosker N was responsible for the manuscript development and approval.

## Figures and Tables

**Table 1:** Patient characteristics.

Age (years)	
Mean (SD)	60.9 (4.7)
Median (Q1; Q3)	60 (57; 64)
Range	54-74
Sex	
Male	352 (49.5%)
Female	359 (50.5%)
Weight (kg)	
Mean (SD)	73.0 (15.2)
Median (Q1; Q3)	71 (62; 81)
Range	35-131
Height (cm)	
Mean (SD)	166.4 (9.1)
Median (Q1; Q3)	167 (160; 173)
Range	141-193
Body mass index (kg/m^2^)	
Mean (SD)	26.3 (4.7)
Median (Q1; Q3)	25.8 (23.3; 28.7)
Range	15.6-49.8
Body surface area (m^2^)	
Mean (SD)	1.83 (0.22)
Median (Q1; Q3)	1.81 (1.67; 1.97)
Range	1.21-2.55

SD: standard deviation.

**Table 2:** Correlations between aortic diameter, age, and body surface area.

Variables	Total	Female	Male
Age	n=711	n=359	n=352
Ascending aorta	0.240 (*p*<0.001)	0.259 (*p*<0.001)	0.172 (*p*=0.001)
Aortic arch	0.252 (*p*<0.001)	0.157 (*p*=0,003)	0.281 (*p*<0.001)
Descending aorta	0.322 (*p*<0.001)	0.190 (*p*<0,001)	0.386 (*p*<0.001)
Body surface area	n=707	n=358	n=349
Ascending aorta	0.350 (*p*<0.001)	0.207 (*p*<0.001)	0.263 (*p*<0.001)
Aortic arch	0.376 (*p*<0.001)	0.288 (*p*<0.001)	0.237 (*p*<0.001)
Descending aorta	0.403 (*p*<0.001)	0.286 (*p*<0.001)	0.203 (*p*<0.001)

Pearson correlation (*p* value).

**Table 3:** Predictors of descending aorta diameter.

Predictor	Unstandardized coefficient (standardized)	Standard error	*p* value
Age	0.108 (0.166)	0.029	<0.001
Male sex	-7.280 (-1.180)	2.479	0.003
Body surface index	3.833 (0.272)	0.491	<0.001
Age and male sex	0.149 (1.494)	0.040	<0.001
Constant	13.811	1.982	<0.001

**Table 4:** Linear regression equations for each segment.

Aortic segment	Linear regression equation
Ascending	**Maximum diameter**=11.849+0.202 x age+3.583 (for male sex)+4.829 × BSA − 0.038 × age (for male sex)
Arch	**Maximum diameter**=15.060+0.095 × age − 4.417 (for male sex)+3.988 × BSA+0.088 × age (for male sex)
Descending	**Maximum diameter**=13.811+0.108 × age − 7.280 (for male sex)+3.833 × BSA+0.149 × age (for male sex)

**Table 5:** Anatomic variations.

Anatomic variations	
Bovine arch	69 (9.7%)
Abnormal origin of vertebral artery	13 (1.8%)
Aberrant right subclavian artery	1 (0.1%)
Aortic arch classification	
Type 1	394 (55.4%)
Type 2	229 (32.2%)
Type 3	88 (12.4%)

## References

[1] Hiratzka LF, Bakris GL, Beckman JA, Bersin RM, Carr VF, Casey DE (2010). 2010 ACCF/AHA/AATS/ACR/ASA/SCA/SCAI/SIR/STS/SVM guidelines for the diagnosis and management of patients with Thoracic Aortic Disease: a report of the American College of Cardiology Foundation/American Heart Association Task Force on Practice Guidelines, American Association for Thoracic Surgery, American College of Radiology, American Stroke Association, Society of Cardiovascular Anesthesiologists, Society for Cardiovascular Angiography and Interventions, Society of Interventional Radiology, Society of Thoracic Surgeons, and Society for Vascular Medicine. Circulation.

[2] Rogers IS, Massaro JM, Truong QA, Mahabadi AA, Kriegel MF, Fox CS (2013). Distribution, determinants, and normal reference values of thoracic and abdominal aortic diameters by computed tomography (from the Framingham Heart Study). Am J Cardiol.

[3] Reed D, Reed C, Stemmermann G, Hayashi T (1992). Are aortic aneurysms caused by atherosclerosis?. Circulation.

[4] Albornoz G, Coady MA, Roberts M, Davies RR, Tranquilli M, Rizzo JA (2006). Familial thoracic aortic aneurysms and dissections--incidence, modes of inheritance, and phenotypic patterns. Ann Thorac Surg.

[5] Elefteriades JA, Farkas EA (2010). Thoracic aortic aneurysm clinically pertinent controversies and uncertainties. J Am Coll Cardiol.

[6] Acosta S, Ogren M, Bengtsson H, Bergqvist D, Lindblad B, Zdanowski Z (2006). Increasing incidence of ruptured abdominal aortic aneurysm: a population-based study. J Vasc Surg.

[7] Svensson LG, Kouchoukos NT, Miller DC, Bavaria JE, Coselli JS, Curi MA (2008). Expert consensus document on the treatment of descending thoracic aortic disease using endovascular stent-grafts. Ann Thorac Surg.

[8] Itani Y, Watanabe S, Masuda Y, Hanamura K, Asakura K, Sone S (2002). Measurement of aortic diameters and detection of asymptomatic aortic aneurysms in a mass screening program using a mobile helical computed tomography unit. Heart Vessels.

[9] Kälsch H, Lehmann N, Möhlenkamp S, Becker A, Moebus S, Schmermund A (2013). Body-surface adjusted aortic reference diameters for improved identification of patients with thoracic aortic aneurysms: results from the population-based Heinz Nixdorf Recall study. Int J Cardiol.

[10] Roman MJ, Devereux RB, Kramer-Fox R, O'Loughlin J (1989). Two-dimensional echocardiographic aortic root dimensions in normal children and adults. Am J Cardiol.

[11] Hartmann LG, Wolosker AM, D'Ippolito G, Borri ML (2001). Angio-RM das artérias carótidas e vertebrais: análise de diferentes técnicas de volume e diluição de contraste em aparelho de 1,0 T e gradiente de 15 mT/m*. Radiol Bras.

[12] Wolak A, Gransar H, Thomson LE, Friedman JD, Hachamovitch R, Gutstein A (2008). Aortic size assessment by noncontrast cardiac computed tomography: normal limits by age, gender, and body surface area. JACC Cardiovasc Imaging.

[13] Lin FY, Devereux RB, Roman MJ, Meng J, Jow VM, Jacobs A (2008). Assessment of the thoracic aorta by multidetector computed tomography: age- and sex-specific reference values in adults without evident cardiovascular disease. J Cardiovasc Comput Tomogr.

[14] Lederle FA, Johnson GR, Wilson SE, Gordon IL, Chute EP, Littooy FN (1997). Relationship of age, gender, race, and body size to infrarenal aortic diameter. The Aneurysm Detection and Management (ADAM) Veterans Affairs Cooperative Study Investigators. J Vasc Surg.

[15] Cho IJ, Jang SY, Chang HJ, Shin S, Shim CY, Hong GR (2014). Aortic aneurysm screening in a high-risk population: a non-contrast computed tomography study in Korean males with hypertension. Korean Circ J.

[16] dos Santos RS, Franceschini JP, Chate RC, Ghefter MC, Kay F, Trajano AL (2016). Do Current Lung Cancer Screening Guidelines Apply for Populations With High Prevalence of Granulomatous Disease? Results From the First Brazilian Lung Cancer Screening Trial (BRELT1). Ann Thorac Surg.

[17] Santo AH, Puech-Leão P, Krutman M (2012). Trends in aortic aneurysm- and dissection-related mortality in the state of São Paulo, Brazil, 1985-2009: multiple-cause-of-death analysis. BMC Public Health.

[18] Mao SS, Ahmadi N, Shah B, Beckmann D, Chen A, Ngo L (2008). Normal thoracic aorta diameter on cardiac computed tomography in healthy asymptomatic adults: impact of age and gender. Acad Radiol.

[19] Hager A, Kaemmerer H, Rapp-Bernhardt U, Blücher S, Rapp K, Bernhardt TM (2002). Diameters of the thoracic aorta throughout life as measured with helical computed tomography. J Thorac Cardiovasc Surg.

[20] Agmon Y, Khandheria BK, Meissner I, Schwartz GL, Sicks JD, Fought AJ (2003). Is aortic dilatation an atherosclerosis-related process? Clinical, laboratory, and transesophageal echocardiographic correlates of thoracic aortic dimensions in the population with implications for thoracic aortic aneurysm formation. J Am Coll Cardiol.

[21] Ergun E, Şimşek B, Koşar PN, Yılmaz BK, Turgut AT (2013). Anatomical variations in branching pattern of arcus aorta: 64-slice CTA appearance. Surg Radiol Anat.

[22] Jalali Kondori B, Asadi MH, Rahimian E, Tahsini MR (2016). Anatomical Variations in Aortic Arch Branching Pattern. Arch Iran Med.

[23] Mylonas SN, Barkans A, Ante M, Wippermann J, Böckler D, Brunkwall JS (2018). Prevalence of Bovine Aortic Arch Variant in Patients with Aortic Dissection and its Implications in the Outcome of Patients with Acute Type B Aortic Dissection. Eur J Vasc Endovasc Surg.

[24] Mongeon FP, Marcotte F, Terrone DG (2016). Multimodality Noninvasive Imaging of Thoracic Aortic Aneurysms: Time to Standardize?. Can J Cardiol.

